# Translation and validation of the simplified Chinese version of the Chronic Pain Coping Inventory-42

**DOI:** 10.1186/s13018-023-03928-w

**Published:** 2023-06-24

**Authors:** Jun Kou, Wei Xu, Qiqi Sun, Qingyun Xie, Wei Wang

**Affiliations:** 1 Department of Orthopedics, General Hospital of Western Theater Command, Rongdu Avenue No. 270, Chengdu, 610000 People’s Republic of China; 2 Department of Ultrasound, Children’s Hospital, Chongqing Medical University, Chongqing, People’s Republic of China; 3 Dean’s Office, West China Hospital, Sichuan University, Chengdu, People’s Republic of China

**Keywords:** Lumbar disc herniation, Translation and adaptation, Chinese, CPCI-42

## Abstract

**Background:**

The Chronic Pain Coping Inventory-42(CPCI-42) is mainly used for chronic pain management project, its original version is written in English and has been widely used in western countries. Therefore, the purpose of our study is to apply the CPCI-42 to Chinese patients and evaluate its responsiveness, reliability, and validity for Chinese patients with lumbar disc herniation (LDH).

**Methods:**

Translation and adaptation were carried out in accordance with the guidelines of the American Academy of Orthopedic Surgeons Outcome Committee. A total of 133 patients who were diagnosed with LDH were included in this study. Psychometric evaluations were then performed and included score distribution, internal consistency, test–retest reliability, construct validity, and responsiveness.

**Results:**

CPCI-42 is well adapted to the assessment of the cognitive and behavioral strategies of patients with LDH, and the scores of score distribution, internal consistency, test–retest reliability, construct validity, and responsiveness are excellent. Forward and reverse translation of the CPCI-42 to English from Chinese worked smoothly.

**Conclusion:**

It is applicable to the assessment of quality of life of the cognitive and behavioral strategies of patients with LDH, and the scores of all indicators are excellent.

## Background

Lumbar disc herniation (LDH) is the most common cause of low back pain. The main signs and symptoms include low back pain, numbness and pain of one or both lower limbs. Focal paralysis, limitation of trunk flexion, and increased leg pain when sitting or coughing and sneezing forcefully may also indicate LDH [1]. The overall incidence rate of LDH is 15–30% in western countries and 18% in China [2], as a populous country, a large number of Chinese people are troubled by the pain caused by LDH. Moreover, the impact of pain caused by LDH on patients is not limited to motor dysfunction, but related to the negative impact on patients' social relations, self-esteem and life satisfaction, or related to psychological disorders such as depression, anxiety and somatoform disorders [3].

The Chronic Pain Coping Inventory-42(CPCI-42) is mainly used for chronic pain management project, which is often used to evaluate the eight types of coping strategies that patients may use to cope with chronic pain, and has been widely used in western countries [4], the Korean [5] and Polish [6] versions of the questionnaire have also been proved to be applicable. In addition, it also shows applicability in assessing chronic pain coping strategies among Hong Kong Chinese [7]; however, this study was conducted in the context of Cantonese speaking, and this measure was verified in Cantonese speaking Hong Kong Chinese. To what extent CPCI-42 can be extended to Chinese people who speak other Chinese dialects (such as Mandarin) is still unknown. In order to solve this problem, we developed a simplified Chinese version of The Chronic Pain Coping Inven-tory-42 (SC-CPCI-42) through cross-cultural adaptation, applied CPCI to people in Chinese Mainland who use Mandarin, and evaluated its reliability and effectiveness in evaluating chronic pain in patients with LDH.

## Methods

### Translation and cross‑cultural adaptation

The English version of CPCI-42 was translated into simplified Chinese in accordance with the principles of the guidelines previously issued [8]. The whole process consists of five steps. The details have been introduced in a similar article published in our previous publication [9]. (1) Forward translation from English to Chinese by 2 bilingual translators independently (an orthopedic surgeon from our department and a professional translator). (2) For a synthesis of the translations, a discussion was held to integrate the two independent forward translation drafts; later we obtained the primary Chinese version of CPCI-42. (3) Backward translation by two independent native English translators who are well conversant in Chinese, the two translators have medical backgrounds, but no knowledge of the original CPCI-42. (4) Creation of a pre-final version: a pre-final version was created by an expert committee after comparing the translated versions to the original version. (5) Twenty patients with LDH were invited to complete the SC-CPCI-42 for assessment, and feedback was collected. A third meeting was conveyed by all research members for final adjustments according to this feedback, and the final version of the SC-CPCI-42 was obtained.

### Patients and data acquisition

We recruited 163 patients with chronic low back pain caused by LDH from outpatients and inpatients of our Hospital. Inclusion criteria are as follows: (1) age > 18, with independent signature right; (2) patients with LDH diagnosed by MRI and the severity is Grade I and Grade II; (3) chronic pain for more than 3 months and/or recurrent back pain with/without radiation pain. The exclusion criteria are as follows: (1) patients with other diseases that cause chronic pain, (2) patients who are receiving conservative treatment or have undergone surgical treatment. All participants read and signed the informed consent form approved by the Ethics Committee. On the first day of admission, patients were asked to provide demographic information and independently completed two scales (SC-CPCI-42, The Wong Baker FACES Pain Rating Scale) in a quiet conference room. One week after the completion of the first set of scales, they completed SC-CPCI-42 for the second time before starting conservative treatment to evaluate the scale of test–retest reliability. If the patient received relevant treatment in the previous week, they will be excluded. Finally, patients receiving 12 weeks of conservative treatment in our hospital completed SC-CPCI-42 for the third time to assess responsiveness. Finally, 133 patients participated in the whole experiment and were included in our analysis.

### Scales

CPCI-42 is a 42 item abbreviated version of CPCI, which evaluates cognitive and behavioral strategies for coping with chronic pain [10]. The 42 items were divided into eight subscales: guarding, resting, asking for assistance, relaxation, task persistence, exercise/stretch, seeking social support and coping self-statement. The Likert style response scale includes 8 levels (0–7), measuring the frequency of participants' using 42 coping strategies, and the number of days they used these strategies to deal with pain at least once in the past week.

The Wong Baker FACES Pain Rating Scale (WBS) uses all five emoticons to assess the range of pain intensity [11], WBS is mainly used to assess the severity of pain in children, and has also been verified in other adults, mainly used to assess chronic pain over the age of three [12].

### Psychometric assessments and statistical analysis

Reliability is the degree to which the measurement is free from error. The reliability test of SC-CPCI-42 mainly includes internal consistency, and test–retest reliability [13]. The degree of internal consistency is derived by the scale of Cronbach’s *α* value of the scale. When *α* > 0.7, 0.8, and 0.9, the scale has acceptable, good, and excellent internal consistency, respectively [9]. Test–retest reliability of the scale was evaluated by comparing with the two responses of patients to SC-CPCI-42. The intraclass correlation coefficient (ICC), based on the two-way analysis of the variance in a random effect model, is its assessment indicator. Once ICC > 0.9 and 0.8, the scale has excellent and good reliability, respectively [14].

The validity of SC-CPCI-42 can be evaluated by its content validity and construct validity. The comprehensiveness and evaluation of the relevance of the items are evaluated by content validity. The comprehensive evaluation indexes of the three items are patients’ feedback, the response rate, and ceiling/floor effects. Assuming that the ceiling/floor effect is less than 15%, the patient's feedback is greater than 95%, and the patient reports no difficulty in filling out the scale, the judgment scale is considered to have great comprehensiveness [15]. The construct validity of the standard was evaluated by calculating the correlation between sc-CPI-42 scale and WBS [16]. The Pearson correlation coefficient represents the relationship between a particular questionnaire score and other validation tools, and the results are expressed as ‘excellent’ (*r* > 0.8), ‘very good’ (r = 0.61–0.80), ‘moderate’ (*r* = 0.41–0.60), ‘fair’ (*r* = 0.21–0.40), and ‘poor’ (*r* < 0.20 or *P* > 0.05).

Responsiveness is a scale used to detect the ability of structures to be measured over a period of time [17]. The results of the scale prior to conservative treatment and 12 weeks after conservative treatment were compared to evaluate the responsiveness of sc-CPI-42. The two indicators of reactivity evaluation are standardized response mean (SRM) and effect size (ES). When the SRM and ES values exceed 0.80, they are large. When the values are between 0.51 and 0.80, they are intermediate, and when they are less than 0.50, they are small [17].

The analyses were performed in SPSS version 25.0 for Windows (Chicago, IL, USA). The data are expressed as the mean and standard deviation (SD). The 95% confidence intervals (CIs) report the ICC value. A P value less than or equal to 0.05 is considered statistically significant.

### Ethical statement

All procedures performed in this study involving human participants were carried out in accordance with the 1964 Helsinki Declaration and its later amendments or comparable ethical standards. All participants read and signed informed consent, and this clinical study obtained the approval of the ethics committee of our hospital.

## Results

### Patients

A total of 163 patients with chronic low back pain caused by LDH met the screening criteria. In the end, 133 patients participated in the whole study. Therefore, 133 samples were selected to evaluate the validity, test–retest reliability, responsiveness, test–retest reliability and internal consistency of SC-CPCI-42. Table 1 shows the detailed demographics data of these participants.

**Table 1:** Demographic and clinical characteristics of participants

Characteristics	Number	No. (%)
Gender (male/female)	101/32	75.94/24.06
*Age (years)*		
Mean ± SD) range	48.47 ± 15.52 18–86	–
*Profession*		
Manual workers	72	54.13
Mental workers	31	23.31
Retirement	30	22.56

*SD* standard deviation

This section may be divided by subheadings. It should provide a concise and precise description of the experimental results, their interpretation, as well as the experimental conclusions that can be drawn.

### Translation and cross‑culture adaptation process

Forward and reverse translation of SC-CPCI-42 worked very smoothly. Since the items of the SC-CPCI-42 are easy to understand, we did not improve them. None of the patients indicated that the project was difficult to understand. There was no lack of standardization.

### Reliability

In this study, there was no error in SC-CPCI-42. The score distribution shows that there is no floor effect or ceiling effect in SC-CPCI-42. According to the correlation strength among 42 items, the internal consistency of SC-CPCI-42 is good, and each item is correlated with its subscale table (Table 2). The ICCs of the total and three aspects are more than 0.9, indicating that the test–retest reliability of SC-CPCI-42 is excellent (Table 3).

**Table 2:** Descriptive statistics, internal consistencies and construct validity of the SC-CPCI-42

Dimension and Scale	Mean (SD)	Cronbach’s *α* (95% CI)	Floor effect	Ceiling effect	r of SC-CPCI-42 with WBS
Guarding	3.063 (1.351)	0.765 (0.698–0.821)	0	0	0.762^*^
Resting	3.723 (1.429)	0.739 (0.661–0.803)	0	0	0.779^*^
Asking for Assistance	2.733 (1.765)	0.881 (0.844–0.911)	0	0	0.607^*^
Relaxation	2.841 (1.365)	0.721 (0.638–0.790)	0	0	0.784^*^
Task Persistence	3.328 (1.517)	0.778 (0.712–0.833)	0	0	0.860^*^
Exercise/Stretch	3.766 (1.491)	0.770 (0.702–0.827)	0	0	0.810^*^
Seeking Social Support	2.809 (1.328)	0.708 (0.622–0.780)	0	0	0.747^*^
Coping Self-Statement	3.206 (1.421)	0.738 (0.660–0.802)	0	0	0.827^*^

*SC-CPCI-42* simplified Chinese version of The Chronic Pain Coping Inventory-42; *SD* standard deviation; *r* Pearson’s correlation coefficient

*P < 0.01

**Table 3:** Test–retest reliability and the responsiveness of the SC-CPCI-42

Dimension and scale	First test Mean ± SD	Second test Mean ± SD	ICC(CI range)	12th week Mean ± SD	12th week SRM	12th week ES
Guarding	3.063 ± 1.351	3.182 ± 1.131	0.904 (0.867–0.931)	2.054 ± 1.279	1.406	0.749
Resting	3.723 ± 1.429	3.731 ± 1.317	0.893 (0.853–0.923)	2.418 ± 1.303	1.719	0.913
Asking for assistance	2.733 ± 1.765	2.838 ± 2.687	0.917 (0.885–0.940)	1.820 ± 1.527	1.265	0.518
Relaxation	2.841 ± 1.365	2.487 ± 1.068	0.885 (0.842–0.917)	1.827 ± 1.263	1.583	0.743
Task persistence	3.328 ± 1.517	3.298 ± 1.339	0.903 (0.866–0.930)	2.183 ± 1.332	1.364	0.754
Exercise/stretch	3.766 ± 1.491	3.600 ± 1.326	0.908 (0.872–0.934)	1.767 ± 1.029	2.236	1.340
Seeking social support	2.809 ± 1.328	2.836 ± 1.189	0.854 (0.800–0.894)	1.765 ± 1.134	1.576	0.786
Coping self-statement	3.206 ± 1.421	3.239 ± 1.274	0.885 (0.842–0.917)	1.898 ± 1.301	1.783	0.921

*SC-CPCI-42* simplified Chinese version of The Chronic Pain Coping Inventory-42; *SD* standard deviation; *ICC* intraclass correlation coefficient

## Validity

There were no errors in the SC-CPCI-42 questionnaire in this study. The score distribution shows that there is no ceiling and floor effect in SC-CPCI-42 score (Table 2). In addition, no patient indicated that the content of SC-CPCI-42 was difficult to understand. After evaluation and analysis by orthopedic and rehabilitation experts in two departments, the amount of information obtained by each SC-CPCI-42 item is sufficient to evaluate the health-related quality of life of patients with chronic low back pain caused by LDH. From the above results, SC-CPCI-42 has good content validity. Table 2 shows the relevant data of ACL-QOL-C construct validity evaluation.

## Responsiveness

The questionnaire was completed before and after conservative treatment to evaluate the responsiveness of SC-CPCI-42. The relevant data are shown in Table 3. Overall, the mean of SC-CPCI-42 score decreased after conservative treatment. After the results of the questionnaire come back in the 12th week, we analyzed the data again and found that, compared with the first time, the SRM and ES of the total score and each item were greater than 0.5, reflecting the high responsiveness of the tool.

## Discussion

Through this study, it is found that SC-CPCI-42 shows good score distribution, high internal consistency, excellent test–retest reliability, and significant structural and discriminant validity. As far as we know, this is the first time to apply CPCI-42 translation, cross-cultural adaptation and research to people in Chinese Mainland. It complements the previous research to a certain extent [7], and applies CPCI-42 to the Chinese population in Mandarin. It has been proved that it can help many Chinese patients with LDH obtain more accurate diagnosis and treatment, and evaluate their coping strategies for chronic pain.

The study have shown that Cronbach *α* are high in the eight projects of SC-CPCI-42, which shows that they have a strong correlation with each other. In the Korean version of the scale, Cronbach α of asking for assistance is relatively low, which may be due to cultural differences among Koreans [5]. Many Koreans prefer to use a heated floor instead of a bed, Chinese people also have the same habit of using beds and sofas to rest as Westerners; therefore, the Cronbach *α* of the Chinese version of asking for assistance in SC-CIPI-42 is relatively high. Good test–retest reliability is reflected in SC-CIPI-42, which is consistent with the results of similar studies. There is no floor effect or ceiling effect in SC-CIPI-42. The evaluation of the three experts also confirmed that the SC-CIPI-42 project is closely related to the prognosis of chronic pain in patients with LDH. In addition, because SC-CIPI-42 is easy to understand, there is no missing reply in any returned questionnaire.

The test–retest reliability evaluation of SC-CIPI-42 was conducted at the interval of one week. The patient is unlikely to remember the specific answers in the previous questionnaire completely, and the functional status and daily life of the patient will not change significantly within one week[9].

In terms of the correlation between the SC-CIPI-42 of WBS, each subfield of SC-CIPI-42 is very good or excellent. WBS expresses pain in an intuitive way, and uses the number range to convey the patient's pain experience in the context of facial expressions[18]. CIPI-42 evaluated the patient's coping strategies for pain, and the SC-CIPI-42 of WBS have a good structural correlation, indicating that SC-CIPI-42 could evaluate the patient's pain well. But the Pearson’s correlation coefficient on Asking for Assistance is lower than that of other subdomains, which may be related to the cultural environment of Chinese people. Most Chinese people express their feelings more implicitly and do not like to turn to others for help[19]. This is the first time that CIPI-42 and WBS have been studied for correlation.

An important factor in determining whether the scale can be used in prospective clinical research is the quality of scale responsiveness [20]. In this study, SC-CIPI-42 showed good responsiveness, which means that SC-CIPI-42 is sensitive to changes in pain coping strategies for patients with LDH after conservative treatment. In previous studies, the responsiveness of CIPI-42 was not studied. Through our research, we found that after a period of conservative treatment, the pain status of patients with LDH was improved to a certain extent, and the quality of life was improved to a certain extent.

According to our results and the data of other language versions, CIPI-42 is well adapted to the assessment of cognitive and behavioral strategies for coping with chronic pain, and the scores of various indicators are excellent. Although there are still some deficiencies in our research, the subjects of our study are all patients with lumbar disc herniation of grade I and II severity, who receive conservative treatment. It needs further study on the evaluation results of the pain of LDH with grade III severity and requiring surgical treatment.

## Conclusions

In short, CIPI-42 was successfully translated into Chinese. After verification, the version was easy to use and shown to have good responsiveness, reliability, and effectiveness. SC-CIPI-42 is well adapted to assess the cognitive and behavioral strategies of patients with LDH for chronic pain.

## Data Availability

The data analyzed for the current study are available from the corresponding author on reasonable request.

## Abbreviations

CPCI-42: The Chronic Pain Coping Inventory-42
LDH: Lumbar disc herniation
SC-CPCI-42: Simplified Chinese version of The Chronic Pain Coping Inven-tory-42
WBS: The Wong Baker FACES Pain Rating Scale
ICC: The intraclass correlation coefficient
SRM: Standardized response mean
ES: Effect size
SD: Standard deviation
CIs: Confidence intervals
